# Tumor-associated macrophages promote cholangiocarcinoma progression via exosomal Circ_0020256

**DOI:** 10.1038/s41419-022-04534-0

**Published:** 2022-01-28

**Authors:** Shaoyi Chen, Zuxiao Chen, Zongyan Li, Shiying Li, Zilong Wen, Liangqi Cao, Yubin Chen, Ping Xue, Haiyan Li, Dawei Zhang

**Affiliations:** 1 Department of pancreatic hepatobiliary Surgery, the Sixth Affiliated Hospital of Sun Yat-sen University, Guangzhou, China; 2 Department of Hepatobiliary Surgery, The Second Affiliated Hospital of Guangzhou Medical University, Guangzhou, China; 3 Department of Breast Surgery, The Sixth Affiliated Hospital of Sun Yat-sen University, Guangzhou, China; 4 Key Laboratory of Molecular Target & Clinical Pharmacology, School of Pharmaceutical Sciences, Guangzhou Medical University, Guangzhou, China

**Keywords:** Cancer, Molecular biology

## Abstract

This study investigated the exosomal circular RNAs (CircRNAs) produced by tumor-associated macrophages and delivered into the microenvironment of cholangiocarcinoma cells in order to use them as molecular targets for clinical therapy. Tumor-associated M2 macrophages (TAMs) were induced from THP-1 cells and identified by flow cytometry. The TAM-secreted exosomes were isolated from conditioned medium and a CircRNA microarray assay was performed to identify CircRNAs that were uniquely expressed in the isolated exosomes. Circ_0020256 was especially identified based on having the highest differential expression level among all of the CircRNA candidates. In vitro *and* in vivo experiments were performed to assess the effects of TAMs, exosomes, and Circ_0020256 on the growth and migration of cholangiocarcinoma (CCA) cells. The induced TAMs promoted the proliferation, migration, and invasion of CCA cells and those effects were mediated by exosomes secreted by the TAMs. In CCA cells (RBE and HCCC-9810), Circ_0020256 significantly promoted cellular activity by interacting with its intra-cellular microRNA target, miR-432-5p. In contrast, overexpression of transcription factor E2F3 in CCA cells restored the CCA cellular activities that were inhibited by miR-432-5p. On the other hand, treatment with small interference RNA (siRNA) for Circ_0020256 inhibited CCA cell proliferation, migration, and invasion both in vitro and in vivo. In conclusion, Circ_0020256 in TAM-secreted exosomes promoted the proliferation, migration, and invasion of CCA cells, and that promotional activity was regulated via a Circ_0020256/miR-432-5p/E2F3 axis.

## Introduction

Cholangiocarcinoma (CCA) is a common primary malignant tumor, and in recent years, its incidence has significantly increased worldwide [1]. CCA comprises a variety of epithelial cancers which are usually diagnosed at a late stage and have a poor prognosis [2]. Surgical resection remains the preferred treatment for CCA, and the median overall survival time of CCA patients who receive chemotherapy is <1 year [3]. Therefore, it is critically important to search for new molecular targets for use in treating patients with CCA.

Tumor-associated macrophages (TAMs) derived from blood monocytes play essential roles in both tumor initiation and progression [4]. Upon stimulation, macrophages can secrete both pro- and anti-inflammatory factors [5]. A previous study in a hamster CCA model showed that a change in the TAMs characteristic of early stage CCA promoted the progression and metastasis of CCA [6]. In addition, a high density of TAMs in surgically resected tumor tissues is predictive of both a high likelihood of CCA recurrence and a poor prognosis [7]. Another recent study showed that the recruitment of PD-L1 + TAMs contributed to CCA progression [8]. Together, these previous findings revealed that TAMs play important roles in CCA progression; however, the molecular mechanisms underlying the effects of TAMs have not yet been identified.

Exosomes are micro-vesicles with a diameter of 30–100 nm and a crucial part of a tumor’s microenvironment [9]. Exosomes contain non-coding RNAs, DNA fragments, and proteins that function as communicators of transduction signals between cancer cells and thus play important roles in tumor development and the drug resistance of tumors [10]. Both tumor cell-derived exosomes and a tumor’s microenvironment were found to promote cancer progression by releasing immunosuppressive cytokines that suppress immune reactions, or by providing tumor cells with substances needed for metabolism [11]. In particular, CCA cell-derived exosomes were found to regulate tumor growth by preventing cytokine-induced killer (CIK) cells from producing tumor necrosis factor [12] or transporting oncogenic proteins that enhance the development and metastasis of CCA [13]. Moreover, human CCA cell-derived exosomes were found to contain higher levels of oncogenic proteins than normal human cholangiocyte-derived exosomes [14]. More importantly, previous studies revealed that exosomes secreted from TAMs affected various cancer-related activities. For example, they were shown to mediate the imbalance of Treg/Th17 cells in epithelial ovarian cancer [15], facilitate hepatocarcinoma metastasis [16], enhance cell migration and invasion in colon cancer [17], and promote the progression of both pancreatic ductal adenocarcinoma [18] and prostate cancer [19]. However, the effects of exosomes secreted by the TAMs of cholangiocarcinoma patients remain unknown.

Circular RNAs (CircRNAs) comprise a class of non-coding RNAs that are highly stable and resistant to exonuclease degradation [20]. Previous studies revealed that CircRNAs play vital roles in cancers, and might serve as biomarkers for use in diagnosing various cancers, including CCA [21]. Several CircRNAs, including CiR-SRY [22], hsa_Circ_0001649 [23], Circ_0005230 [24], CircRNA Cdr1as [25], Circ-SMARCA5 [26], and Circ-CCAC1 [27] have been found to regulate the progression of CCA and serve as potential biomarkers for CCA. Those previous findings suggested that CircRNAs might act as major regulators of CCA, as well as potential therapeutic targets for CCA. In particular, recent findings showed that Circ-0000284 transmitted by exosomes can competitively bind to miR-637 and induce CCA progression by modulating the expression of lymphocyte antigen six family member E (LY6E) [28]. This further suggested that exosomal CircRNAs play a role in CCA progression.

In this study, we performed a CircRNA microarray assay to identify CircRNAs that were differentially expressed in exosomes secreted by TAMs. Next, the identified CircRNA candidates were tested for their abilities to promote CCA cell proliferation, migration, and invasion both in vitro and in vivo. When compared with all other identified CircRNA candidates from TAM-derived exosomes, Circ_0020256 showed much higher levels of those promotion abilities. Our subsequent studies focused on how Circ_0020256 might regulate CCA progression and the potential use of Circ_0020256 as a molecular target for clinical therapy.

## Materials and methods

### Clinical characteristics of the patients

Clinical information regarding the human CCA specimens is summarized in Supporting Table 1. Briefly, the patients had a medium age of 60.5 ± 6.4 years and 19 (63.33%) of them were male. There were 19 (63.33%) cases of hilar cholangiocarcinoma and 11 (36.67%) cases of distal cholangiocarcinoma; 20 (66.67%) cases were accompanied by vascular invasion. All patients had lymph node metastasis. TNM (tumor, node, metastasis) stage was determined using the American Joint Committee on Cancer/International Union Against Cancer tumor classification system, with 18 patients classified as T3 stage and 12 patients as T4 stage. The distribution of tumor differentiation was as follows: 11 cases were well differentiated; eight cases were moderately differentiated; 11 cases were poorly differentiated.

### Cell culture and clinical specimens

Human monocyte THP-1 cells, HEK293 cells, and human CCA cell lines HCCC-9810 and RBE (National Collection of Authenticated Cell Cultures, Shanghai, China) were cultured in RPMI-1640 medium supplemented with 10% fetal bovine serum at 37 ^°^C in a 5% CO_2_ atmosphere. Clinical specimens (including tumor tissues and paracancerous tissues) from 30 patients were obtained from individuals who had been originally diagnosed with hepatobiliary cancer in the clinic, subsequently had their tumors confirmed pathologically as CCA, and later underwent curative resection at the Second Affiliated Hospital of Guangzhou Medical College (Guangzhou, China) between March 2016 and April 2018. Pertinent patient clinical reports were obtained with prior patient consent and the approval of the institutional Clinical Ethics Review Board.

### THP-1 differentiation and exosome stimulation

THP-1 cells were treated with PMA (100 ng/mL) for 24 h to induce macrophage differentiation. Suspended THP-1 cells were treated with PMA to induce their differentiation; after which, a large majority (>95%) of the treated cells adhered to the dish. Next, the cells were treated with 20 ng/mL IL-4 during a 24 h polarization period. After being washed twice with PBS, the cells were treated with 200 ng/mL TWEAK for 24 h to stimulate the secretion of exosomes.

### Exosome extraction from cell culture conditioned medium (CM) and identification by western blotting

Exosomes were extracted using ExoQuick™ precipitation solution according to the manufacturer’s instructions. Briefly, 500 µL of CM cells were mixed with 1 mL of ExoQuick-TC™ solution and incubated overnight at 4 °C. After centrifugation, the pellet was resuspended in 200 µL of PBS and purified using columns. The exosomes were eluted with 200 µL of PBS and subsequently identified by their expression of CD63, TSG101, and CD9 antigens.

### CircRNA microarray assay

Exosomes isolated from the supernatants of M2 macrophages (control: THP-1 cells were treated with PMA and TWEAK; test: THP-1 cells were stimulated with PMA + IL-4 and TWEAK) were collected and their total RNA was extracted using Trizol reagent. The amount of total RNA was determined by capillary electrophoresis performed on an Agilent 2100 Bioanalyzer. CircRNA microarray assays were performed by Shanghai Biotechnology Corporation. Human CircRNA Array v2 was designed with four identical arrays per slide, with each array containing probes that interrogated ~170,340 human circRNAs.

### In vivo xenograft assay

BALB/c nude mice (6 weeks old) were housed at Guangzhou Medical University. All experimental protocols were approved by the Institutional Animal Care and Use Committee of Guangzhou Medical University. The mice were randomly assigned to four groups, *n* = 5 per group: control (RBE or HCCC-9810 cells only), exosomes (Exo), Circ_0020256 (Circ_X), and exosomes + Circ_0020256 siRNA (Exo + siCirc_X). A total of 1 × 10^7^ cells were subcutaneously injected into each mouse, and the body weight of each mouse was measured once a week. After 4 weeks, the mice were sacrificed and the tumors were removed. Tumor volume was calculated as: 0.5 × width^2^ × length.

### Statistical analysis

All statistical analyses were performed using the Student’s *t*-test or one-way analysis of variance. Each experiment has been repeated a minimum of three times. The experimental data were summarized using SPSS 17.0 software. Differences with a *p*-value < 0.05 were considered to be statistically significant.

Additional materials and methods are described in the Supporting information. SiRNA, miRNA mimics, and primers used in the study are shown in Supporting tables 2, 3.

## Results

### Tumor-associated macrophages (TAMs) promoted the proliferation, migration, and invasion of CCA cells

**Fig. 1:**
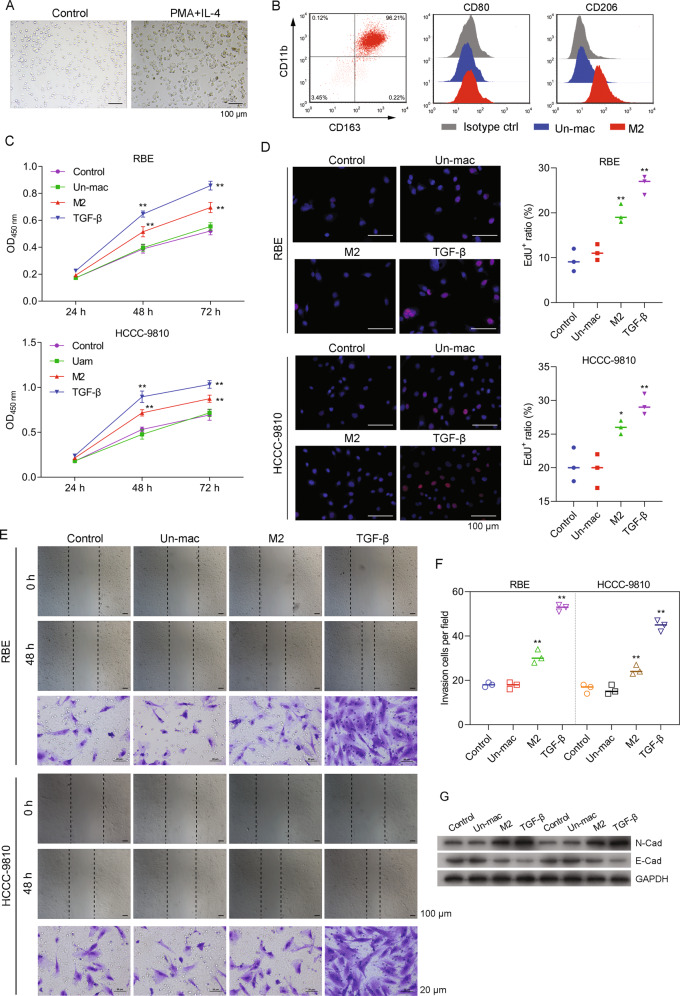
TAMs promoted CCA cell proliferation, migration, and invasion. **A** THP-1 cells were treated with PMA (100 ng/mL) + IL-4 (20 ng/mL) to induce macrophage differentiation. **B** Differentiated THP-1 cells were collected and stained with the indicated antibodies for subsequent flow cytometry analysis. **C** RBE cells (upper panel) and HCCC-9810 (lower panel) cells were co-cultured with M2 macrophages (M2), untreated THP-1 cells (Un-mac), or treated with TGF-β. After 24, 48, and 72 h, CCK8 solution was added to the cells and the absorbance at 450 nm was measured. **D** The respective images of Edu staining assays and a summary of results from three different experiments are shown. **E** Cell monolayers were wounded and images were acquired at 0 and 48 h. The respective images of Transwell assays are shown (lower panel). **F** Results of cell invasion assays as summarized from three independent experiments. **G** Cells were treated as described above and lysed for western blot assays. *p < 0.05 and **p < 0.01 compared with the control group.

THP-1 cells, as a model of monocytes differentiating into macrophages, were used in our studies in order to illustrate the effects of TAMs on both the progression and metastasis of CCA. Prior to PMA and IL-4 treatment, the THP-1 cells displayed a round shape and were suspended in culture. After induction with PMA and IL-4, however, they differentiated into M2 macrophages that adhered to culture the plates (Fig. 1A). The presence of differentiated M2 macrophages was further verified by flow cytometry assays that used CD11b, CD163, CD80, and CD206 antibodies as markers of M2 macrophages (Fig. 1B). Next, the proliferation of cultured CCA cells (both RBE and HCCC-9810 cells) was analyzed after the cells had been transferred to M2 macrophage-conditioned medium (CM). Results of cell expansion studies indicated that both types of CCA cells cultured in CM showed increased rates of proliferation when compared to CCA cells in control medium or CCA cells co-cultured with untreated THP-1 cells (Un-mac) (Fig. 1C). Those results were further supported by the increased proliferation of CCA cells cultured in a medium containing TGF-β as a positive control. In addition, EdU staining assays further proved that CM significantly induced the proliferation of CCA cells (Fig. 1D). Moreover, both types of CCA cells cultured in CM also showed significant increases in their rates of migration and invasion (Fig. 1E, F, Supporting Fig. 1). Consistent with those findings, both types of CCA cells cultured in CM showed enhanced expression of N-cadherin and reduced levels of E-cadherin expression (Fig. 1G); both of which are proteins involved in the EMT process. When taken together, our results suggested that our TAMs, as models of induced M2 macrophages, released uncharacterized factors into the culture medium that promoted the proliferation, migration, and invasion of CCA cells.

### TAM-secreted exosomes induced the proliferation, migration, and invasion of CCA cells

**Fig. 2:**
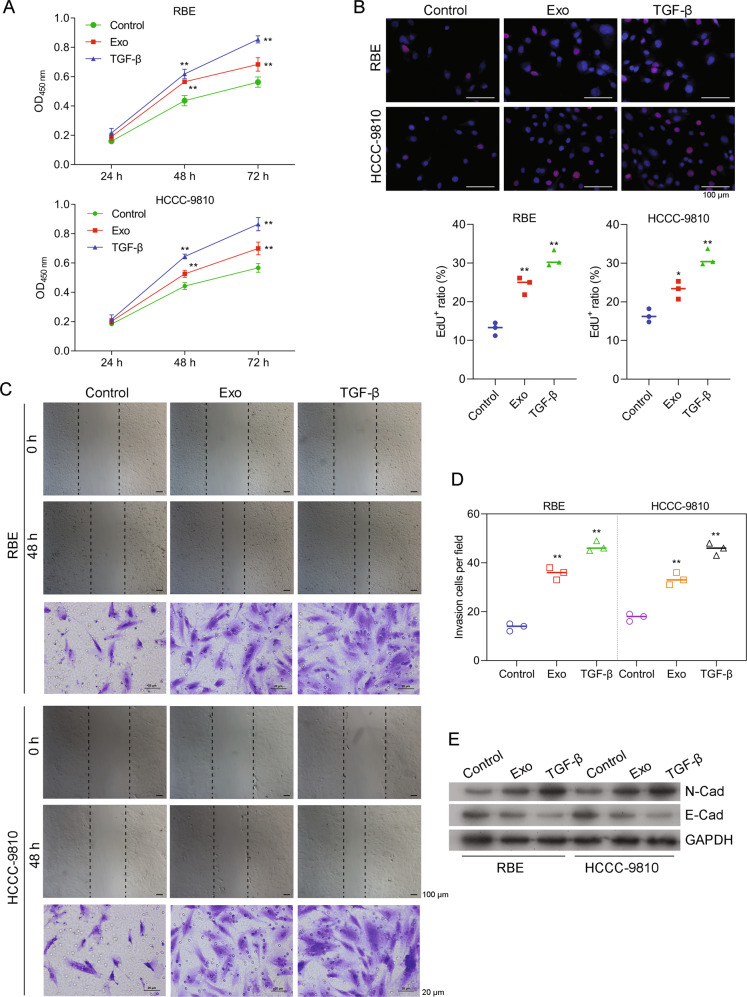
TAM-secreted exosomes induced CCA cell proliferation, migration, and invasion. **A** THP-1 cells were treated with PMA for 24 h and then with IL-4 for the next 24 h to induce macrophage differentiation. The cells were then washed and treated with 200 ng/mL TWEAK for 24 h to release exosomes. Exosomes were extracted from the CM of TAMs. RBE (upper panel) and HCCC-9810 (lower panel) cells were co-cultured with exosomes (Exo) or treated with TGF-β. Cell proliferation was measured by the CCK8 assay. **B** Representative images of Edu staining assays and a summary of results from three different experiments are shown. **C** Cell monolayers were wounded and images were acquired at 0 and 48 h. The respective images of Transwell assays are shown (lower panel). **D** The results of cell invasion assays as summarized from three independent experiments. **E** Cells were treated as described above and then lysed for western blot assays. *p < 0.05 and **p < 0.01 compared with the control group.

Because both the purified exosomes and exosomal contents were found to act as cellular mediators of interactions and communications between tumor cells and macrophages [29], we hypothesized that the above TAM-promoted activities of CCA cells might be regulated by TAM-secreted exosomes. First, micro-vesicles were isolated from the CM of M2 macrophages, which were morphologically characterized by a TEM imaging assay. The mico-vesicles were found to have diameters of 30–150 nM, which was consistent with previously reported features of exosomes (Supporting Fig. 2A). Next, their expression of typical microsomal biomarkers was verified by western blotting (Supporting Fig. 2B). In addition, results of CCK8 assays clearly showed that the CM-isolated exosomes could significantly increase the levels of cell expansion (Fig. 2A). EdU staining assays further confirmed that the increases in cell expansion induced by CM-isolated exosomes were caused by increases in cell proliferation (Fig. 2B). Moreover, wound healing and Transwell assays showed that the CM-isolated exosomes also significantly enhanced the migration and invasion capabilities of CCA cells (Fig. 2C, D and Supporting Fig. 2C). Furthermore, the expression of EMT markers (E-cadherin and N-cadherin) was significantly regulated by the CM-isolated exosomes (Fig. 2E). When taken together, these results confirmed that the CM-isolated exosomes that were originally secreted from TAMs could induce the proliferation, migration, and invasion of CCA cells, suggesting that CM-isolated exosomes mediated the malignancy of CCA.

### Differentially expressed CircRNAs in the TAM-secreted exosomes

**Fig. 3:**
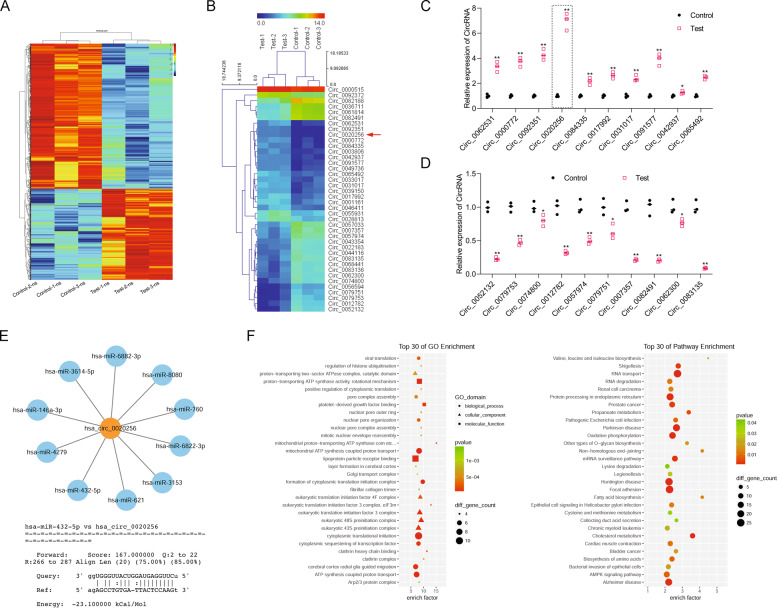
Existence of differentially expressed CircRNAs in the TAM-secreted exosomes. **A** RNA was extracted from the exosomes secreted by TAMs. Differentially expressed genes are shown by a heatmap. Totals of 495 upregulated CicrRNAs and 789 downregulated CircRNAs were detected. ns, normalized signal. **B** The top 20 upregulated and downregulated CircRNAs are shown. **C**, **D** CircRNAs that were upregulated or downregulated in supernatant exosomes were identified by real-time PCR (control: PMA- and TWEAK-treated THP-1 cells, test: PMA + IL-4 and TWEAK-treated cells). **E** Based on a bioinformatics analysis of targeted binding miRNAs (predicted binding energy), ten miRNAs with the highest binding free energy scores were selected (upper panel). MiR-432-5p had the highest binding energy for hsa_Circ_0020256 (lower panel). **F** Top 30 of GO enrichment top 30 of pathway enrichment were shown.

In order to investigate the underlined mechanism by which TAM-secreted exosomes affected CCA tumor progression, a CircRNA microarray assay was performed to identify CircRNAs that were differentially expressed in TAMs-secreted exosomes (*p* < 0.05, fold-change > 2.0, Fig. 3A, B). The numbers of differentially expressed circRNAs are shown in Supporting tables 4, 5. To confirm the results from CircRNA microarray, we detected the expression of differentially expressed CircRNA by real-time PCR (Fig. 3C, D) and found that hsa_Circ_0020256 was one of the upregulated CircRNAs in TAM-secreted exosomes (Fig. 3C). Bioinformatics analysis revealed potential sites for binding between hsa_Circ_0020256 and several different miRNAs, including miR-432-5p, miR-3614-5p, miR-6882-3p, miR-8080, miR-760, miR-3153, miR-621, miR-4279, and miR-146a-3p. Remarkably, the potential binding sites and free energy scores for both hsa_Circ_0020256 and miR-432-5p were specifically identified (Fig. 3E). Next, GO and KEGG pathway enrichment analyses, which are usually performed to characterize the functions of differentially expressed CircRNAs and their involved signaling pathways, revealed that hsa_Circ_0020256 was significantly correlated with growth factor binding and a cancer progression pathway (Fig. 3F). These data suggested that hsa_Circ_0020256 might be involved in the proliferation and migration of CCA cells, as well as the process of CCA development.

### Hsa_Circ_0020256 was highly expressed in TAM-secreted exosomes and regulated the proliferation, migration, and invasion of exosome-enhanced CCA cells

**Fig. 4:**
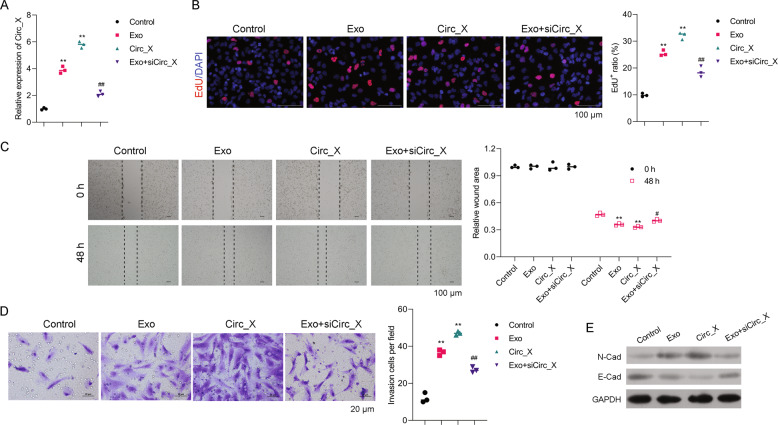
Hsa_Circ_0020256 was highly expressed in TAM-secreted exosomes and regulated the exosome-enhanced proliferation, migration, and invasion of CCA cells. **A** RBE cells were co-cultured with exosomes (Exo), transfected with hsa_Circ_0020256 (Circ_X) or both treated with exosomes and transfected with hsa_Circ_0020256 siRNA (Exo + siCirc_X). **B** Hsa_Circ_0020256 expression was detected by real-time PCR. The respective images of Edu staining assays and a summary of results from three different experiments are shown. **C** Cell monolayers were wounded and images were acquired at 0 and 48 h. **D** The respective images of Transwell assays are shown (lower panel). The results of cell invasion assays as summarized from three independent experiments. **E** Cells were treated as described above and lysed for western blot assays. **p < 0.01 compared with the Control group. ^#^*p* < 0.01 and ^##^*p* < 0.01 compared with the Exo group.

As detected by real-time PCR, we found that hsa_Circ_0020256 expression was increased in RBE cells co-cultured with TAM-secreted exosomes, when compared to control cells without exosome treatment (Fig. 4A). Results from EdU staining, wound healing, and Transwell assays revealed that CCA cell proliferation, migration, and invasion were all significantly enhanced by treatment of co-culturing RBE cells with TAM-secreted exosomes, or by overexpression of hsa_Circ_0020256 in RBE cells (Fig. 4B–D). However, suppression of hsa_Circ_0020256 by siRNA reversed the increased levels of RBE cell proliferation, migration, and invasion among RBE cells that were treated with TAM-secreted exosomes (Fig. 4B–D). Moreover, the levels of E-cadherin, as an EMT marker, were also enhanced by treatment with TAMs-secreted exosomes (Fig. 4E). When taken together, the above results suggested that TAM-secreted exosomes promoted CCA cell proliferation, migration, and invasion via hsa_Circ_0020256.

### Hsa_Circ_0020256 enhanced CCA cell proliferation, migration, and invasion via specific miR-432-5p sponging

**Fig. 5:**
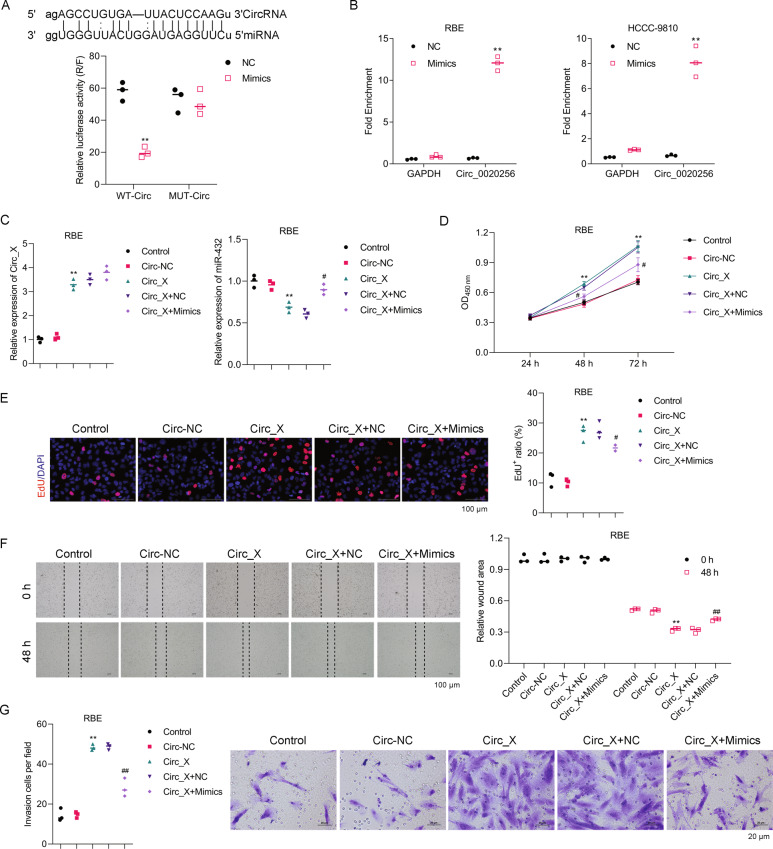
Hsa_Circ_0020256 enhanced CCA cell proliferation, migration, and invasion via specific miR-432-5p sponging. **A** The binding sites for hsa_Circ_0020256 on miR-432-5p (upper panel). HEK293T cells were co-transfected with wild-type hsa_Circ_0020256 (WT-Circ) and the NC or miR-432-5p mimics (mimics), or the mutant form of hsa_Circ_0020256 (MUT-Circ) the and NC or miR-432-5p mimics (mimics), and relative luciferase activity was measured. Summary results for luciferase activity assays are shown (lower panel). **B** The miRNA target IP results from assays performed with HCCC-9810 and RBE cells. **C** RBE cells were transfected with hsa_Circ_0020256 (Circ_X) or co-transfected with hsa_Circ_0020256 and the NC (Circ + NC) or miR-432-5p mimics (Circ_X + mimics). Hsa_Circ_0020256 and miR-432-5p expression were detected by real-time PCR. **D** The CCK8 assay results are shown. **E** The respective images of Edu staining results and a summary of results from three different experiments are shown. **F** Cell monolayers were wounded and images were acquired at 0 and 48 h. The respective images of Transwell assays are shown. **G** The results of cell invasion assays as summarized from three independent experiments. *p < 0.05 and **p < 0.01 compared with the Circ-NC group. ^#^*p* < 0.05 and ^##^*p* < 0.01 compared with the Circ_X + NC group.

A bioinformatics assay revealed that microRNA miR-432-5p has several binding sites complementary to those of hsa_Circ_0020256 (Fig. 5A, upper panel). Additionally, treatment with miR-432-5p mimics significantly reduced the luciferase activity of wild-type hsa_Circ_0020256, while that activity was not changed by treatment with the mutant form of hsa_Circ_0020256 (Fig. 5A, lower panel). The specific binding between miR-432-5p and hsa_Circ_0020256 was further confirmed by miRNA Target IP assays performed with both HCCC-9810 and RBE cells (Fig. 5B). Moreover, our results showed that miR-432-5p was downregulated in Circ_0020256-treated cells, while treatment with miR-432-5p mimics restored the downregulated expression of miR-432-5p (Fig. 5C and Supporting Fig. 3A). Thus, miR-423-5p could be regarded as a specific sponger of Circ_0020256.

Next, overexpression of hsa_Circ_0020256 was established in both RBE and HCCC-9810 cells and was found to significantly upregulate the proliferation of both cell types (Fig. 5D, E and Supporting Fig. 3B, C). However, the hsa_Circ_0020256 overexpression-induced upregulation of cell proliferation induced in both cell types was reversed by the miR-432-5p mimics (Fig. 5D, E and Supporting Fig. 3B, C). In addition, CCA cell migration and invasion were also upregulated in both types of cells after hsa_Circ_0020256 was overexpressed (Fig. 5F, G and Supporting Fig. 3D, E), while miR_432-5p reversed those upregulation effects, as shown by results of wound healing and Transwell assays (Fig. 5F, G and Supporting Fig. 3D, E).

All of the above data suggested that hsa_Circ_0020256 could enhance CCA cell proliferation, migration, and invasion. However, miR-432-5p reversed the upregulation of those activities, indicating that miR-432-5p was a specific sponger of hsa_Circ_0020256.

### Transcription factor E2F3 was identified as a downstream target of miR-43-5p

**Fig. 6:**
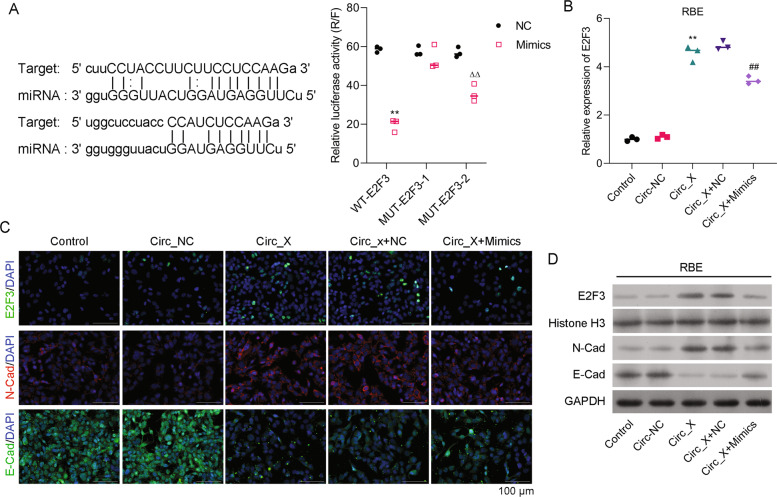
Transcription factor E2F3 was a downstream target of miR-43-5p after interaction of hsa_Circ_0020256/miR-432-5p. **A** The binding sites in the 3'UTR of E2F3 on miR-432-5p (left panel). HEK293T cells were co-transfected with the wild-type E2F3 (WT-E2F3) and NC or miR-432-5p mimics (mimics), or with the mutant form of E2F3 (MUT-E2F3) and NC or miR-432-5p mimics (mimics), and the relative luciferase activity was summarized (right panel). **B** RBE cells were transfected with hsa_Circ_0020256 (Circ_X) or co-transfected with hsa_Circ_0020256 and the NC (Circ + NC) or miR-432-5p mimics (Circ_X + mimics). E2F3 expression was detected by real-time PCR. **C, D** The levels of E2F3, N-Cadherin, and E-Cadherin protein expression were determined by IF or western blotting. **p < 0.01 compared with the Circ-NC group. ^ΔΔ^*p* < 0.01 compared with the NC group. ^##^*p* < 0.01 compared with the Circ_X + NC group.

Next, we searched for potential downstream targets of miR-43-5p. A bioinformatics analysis indicated that the 3' UTR of E2F3 had two potential binding sites (95-102 and 1487–1493) for miR-432-5p (Fig. 6A, left panel). Results of luciferase assays showed that miR-432-5p mimics significantly suppressed the transcriptional activity of E2F3 with the wild-type promoter, but not the transcriptional activity of E2F3 with the mutant promoter (Fig. 6A, right panel), suggesting that E2F3 is a direct target of miR-432-5p. Moreover, real-time PCR results indicated that overexpression of hsa_Circ_0020256 significantly upregulated E2F3 mRNA expression, and that effect could be reversed by the miR-432-5p mimics (Fig. 6B and Supporting Fig. 4A). Results of both IF and western blot assays suggested that E2F3 protein expression was also regulated by hsa_Circ_0020256 overexpression, as well as the interaction between hsa_Circ_0020256 and miR-432-5p (Fig. 6C, D and Supporting Fig. 4B, C). Furthermore, hsa_Circ_0020256 overexpression induced both a downregulation of E-cadherin expression and an upregulation of N-Cadherin, and both of those effects could be reversed by the miR-432-5p mimics (Fig. 6C, D and Supporting Fig. 4B, C). The above results suggested that E2F3 might be a downstream target of miR-43-5p.

### The miR-432-5p/E2F3 axis was involved in regulating CCA cell proliferation, migration, and invasion

**Fig. 7:**
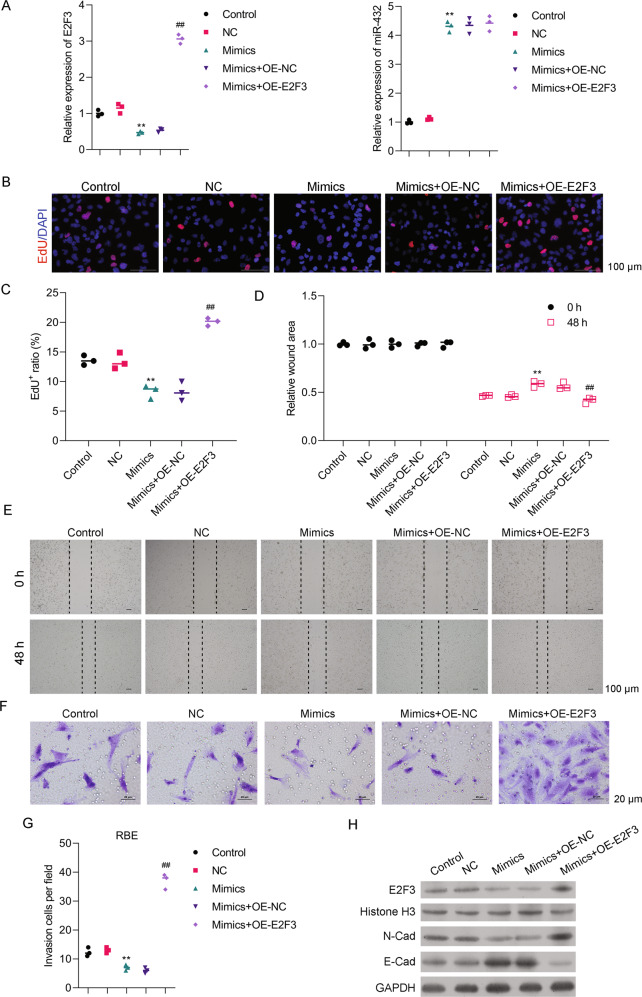
An miR-432-5p/E2F3 axis was involved in regulating CCA cell proliferation, migration, and invasion. RBE cells were transfected with miR-432-5p mimics (mimics) or co-transfected with miR-432-5p mimics and a vector control (mimics + OE-NC) or an E2F3 overexpression plasmid (mimics + OE-E2F3). **A** E2F3 and miR-432-5p mRNA expression were detected by real-time PCR. **B** The respective images of Edu staining results. **C** The summary results of three different experiments are shown. **D** The wound healing results are shown. Cell monolayers were wounded and images were acquired at 0 and 48 h. **E** The respective images of wound healing assays are shown. **F** The wound healing assay results are shown. **G** The results of cell invasion assays as summarized from three independent experiments. **H** Western blot assays were performed using the indicated antibodies. **p < 0.01 compared with the NC group. ^##^*p* < 0.01 compared with the mimics + OE-NC group.

To understand how the targeting of E2F3 by miR-432-5p affects CCA progression, the interaction between miR-432 mimics and E2F3 was analyzed in RBE cells. The levels of miR-432 and E2F3 mRNA expression were detected by PCR (Fig. 7A). Results of EdU staining assays showed that cell proliferation was inhibited by transfection with the miR-432-5p mimics (Fig. 7B), but that effect of miR-432-5p mimics could be reversed by overexpression of E2F3 (Fig. 7B, C). Similarly, the miR-432-5p-induced reductions in cell migration and invasion were also restored by overexpression of E2F3 (Fig. 7D–G). Moreover, the levels of both N-cadherin and E-cadherin expression were regulated by the miR-432-5p/E2F3 (Fig. 7H). These results indicated that a Circ_0020256/miR-432-5P/E2F3 axis was involved in regulating CCA cell proliferation, migration, and invasion.

### Exosomal Hsa_Circ_0020256 mediated TAM-induced tumor progression in vivo via the miR-432-5p/E2F3 axis

**Fig. 8:**
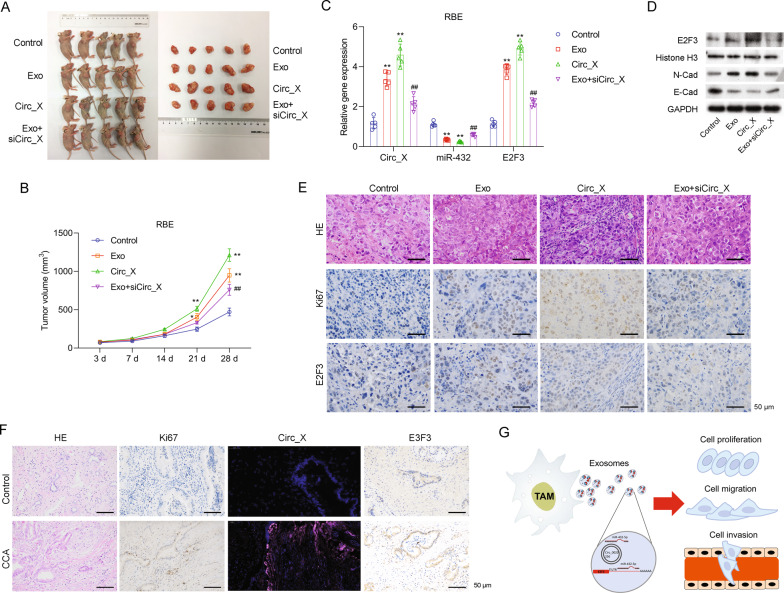
Exosomal Hsa_Circ_0020256 mediated TAM-induced tumor progression in vivo via the miR-432-5p/E2F3 axis. RBE cells (1 × 10^7^) were subcutaneously injected into nude mice. **A** The nude mice were sacrificed after 4 weeks. Images of the xenografts are shown. **B** Tumor volumes were calculated each week and a tumor growth curve is shown. **C** Xenograft tumor tissues were collected and the RNA was extracted. Hsa_Circ_0020256, miR-432-5p, and E2F3 expression were detected by real-time PCR. **D** E2F3, N-Cadherin, and E-cadherin protein expression in xenograft tumor tissues was detected by western blotting. **E** Tumor tissues were collected and fixed for H&E staining and IHC was performed using Ki67 and E2F3 antibodies. **F** Tumor tissues and paracancerous tissues were fixed for FISH assays, H&E staining, and IHC performed using Ki67 and E2F3 antibodies. **G** A schematic model showing how Hsa_Circ_0020256 in TAM-secreted exosomes induces the proliferation, migration, and invasion of CCA cells by sponging miR-432-5p/E2F3.

We next conducted in vivo studies in nude mice with xeno-engrafted CCAs (RBE and HCCC-9810 cells were subcutaneously injected to nude mice respectively) to determine whether TAM-secreted exosomes could promote tumor growth. Remarkably, inhibition of hsa_Circ_0020256 reduced the increases in tumor volume caused by TAM-secreted exosomes (Fig. 8A, B and Supporting Fig. 5A, B). In addition, we investigated the levels of hsa_Circ_0020256, miR-432-5p, and E2F3 expression in the xenograft tissues. As shown in Fig. 8C and Supporting Fig. 5C, treatment with TAM-secreted exosomes significantly increased the levels of both hsa_Circ_0020256 and E2F3 expression, but significantly decreased miR-432-5p expression, while inhibition of hsa_Circ_0020256 reversed those effects. In addition, our data showed that N-cadherin and E-cadherin expression in the cells of xenografted CCA tumor tissues were also regulated by both TAM-secreted exosomes and hsa_Circ_0020256 (Fig. 8D and Supporting Fig. 5D). Moreover, both TAM-secreted exosomes and hsa_Circ_0020256 induced increases in the numbers of Ki67 and E2F3 positive cells in xeno-engrafted CCA tumor tissues, while hsa_Circ_0020256 siRNA reversed those increases (Fig. 8E and Supporting Fig. 5E). These data suggested that hsa_Circ_0020256 was involved in TAM-secreted exosome-induced tumor growth in vivo. Moreover, results of a FISH analysis showed that hsa_Circ_0020256 was mainly expressed in proliferating cells that were positive for Ki67 immunostaining, and the levels of Circ_0020256 in proliferating cells were significantly higher in the clinical tumor tissues of CCA patients than in their paracancerous tissues (Fig. 8F). Furthermore, the levels of E2F3 expression were also significantly higher in the clinical CCA tumor tissues than the paracancerous tissues (Fig. 8F). More importantly, we examined the levels of Circ_0020256 expression in CCA patients and found that Circ_0020256 was expressed at significantly higher levels in the tumor tissues than in the control tissues. (Supporting Fig. 6A). Additionally, the overall survival time and recurrence time of CCA patients was found to be negatively correlated with Circ_0020256 expression (Supporting Fig. 6B, C). When taken together, these results indicated that an hsa_Circ_0020256/miR-432-5p/E2F3 axis plays a role in CCA progression.

## Discussion

Exosomal miRNAs have recently emerged as mediators of crosstalk between tumor cells and macrophages. In this study, the exosomes secreted by PMA- and IL-4-differentiated TAMs were found to significantly increase CCA tumor progression as determined by using both in vivo and in vitro parameters. In vitro studies showed that TAM-secreted exosomes significantly enhanced the proliferation, migration, and invasion of CCA cells, potentially via an hsa_Circ_0020256/miR-432-5p-E2F3 axis. TAM-secreted exosomes also significantly enhanced various parameters known to be related to CCA progression in a xenograft mouse model. Our findings increase our understanding of how TAMs may enhance the progression of CCA via CircRNA-miRNA interactions.

CCA is an aggressive malignancy with a poor prognosis and high mortality rate [30]. Various cell types in the tumor’s microenvironment can affect the progression and metastasis of CCA; those cell types include TAMs, neutrophils, regulatory T lymphocytes (Tregs), and natural killer cells [31]. High infiltration of TAMs is related to a poor clinical outcome for CCA patients [32]. Recent studies have shown that TAMs accelerate CCA cell proliferation by activating the Wnt/β-catenin pathway [33, 34]. Moreover, a high number of TAMs and recruitment of PD-L1 + TAMs contribute to CAA progression and a poor prognosis [7, 8]. Here, we showed that TAM-secreted exosomes isolated from the CM of PMA- and IL-4-differentiated TAMs significantly enhanced the proliferation, migration, and invasion of CCA cells, and also increased the expression of EMT markers in CCA cells. These findings were consistent with previous results (reference here), and further extend our understanding of how TAMs play important roles in CCA progression.

As major players in the tumor microenvironment, TAMs help to regulate tumor angiogenesis, invasion, and metastasis by releasing different types of factors [35]. Extensive evidence suggests that TAM-secreted exosomes play important roles in cell–cell communications [36]. Previous studies have shown that TAM-secreted exosomes regulate the invasiveness of breast cancer [37]. promote the migration of gastric cancer cells [38], and confer cisplatin resistance in gastric cancer cells [39]. TWEAK binds to the Fn14 receptor in TAM-secreted exosomes, and thereby activates several signaling pathways and promotes tumor progression [40]. A recent study revealed that TWEAK-stimulated macrophages affect the metastasis of epithelial ovarian cancer cells via exosomal miR-7 [16]. In the present study, we showed that TAM-secreted exosomes promoted CCA cell proliferation, migration, and invasion in vitro and progression of CCA in vivo, suggesting that TAM-secreted exosomes might be major mediators of crosstalk between TAMs and CCA cells.

CircRNAs are known to be dysregulated in several types of cancer, including CCA [23]. The CircRNA CDR1AS is significantly expressed in CCA tumor tissues and known to be associated with lymph node invasion, an advanced TNM stage, and postoperative cancer recurrence [25]. Several other CircRNAs are also dysregulated in CCA cells and CCA tumor tissues and are associated with tumor size and the differentiation grade of CCA [22–27]. All the above known upregulated or downregulated CircRNAs are expressed in cells or tumor tissues. However, RNA-seq analyses have shown that CircRNAs are also enriched in exosomes [21]. A recent study revealed that the levels of Circ-0000284 were elevated in the exosomes secreted by CCA cells [28]. Here, by using the CircRNA microarray assay, we found that hsa_Circ_0020256 was highly expressed in TAM-secreted exosomes. Further studies revealed that the cellular effects mediated by TAM-secreted exosomes were due to an upregulation of hsa_Circ_0020256 levels, suggesting that hsa_Circ_0020256, as a previously unknown CircRNA member, was responsible for enabling TAM-secreted exosomes to induce CCA progression.

CircRNAs function as post-transcriptional regulators by acting as microRNAs (miRNAs) or protein inhibitors (“spongers”) [41]. CircRNAs have been shown to competitively bind to miRNA, leading to the inhibition of miRNA and their target mRNA molecules [42]. In cancers, many CircRNAs act as competitive inhibitors of miRNAs to regulate the function or translation of target genes. This function allows CirRNAs to play important roles in cancer progression by mediating tumor cell proliferation, migration, and metastasis [43]. In this study, we found that miR-432-5p mimics inhibited hsa_Circ_0020256-induced CCA cell proliferation, migration, and invasion, indicating that miR-432-5p was a sponger of hsa_Circ_0020256. Several studies have shown that miR-432-5p is regulated by CircRNAs in cancer cells and modulates cell growth and metastasis [44–46]. MiR-432-5P has been identified as a target of hsa_Circ_0008039 or circFOXO3, and to play inhibitory roles in breast cancer [44] and glioblastoma [45]. In addition, as a sponger of linc00668, miR-432-5P acts on the EMT process to facilitate the anti-migration function of A549 cells under conditions of an inflammatory microenvironment [47]. Furthermore, miR-432-5p inhibits cell migration and invasion by targeting CXCL15 in colorectal cancer [48]. Here, our findings revealed the effect of miR-432-5p on CCA progression. MiRNAs usually play regulatory roles by pairing with certain mRNAs to cause their post-transcriptional repression [49]. Transcription factor E2F3 plays critical roles in cell proliferation, cell cycle distribution, and cell apoptosis in both tumor and primary cells [50] by acting as a target for various miRNAs (including miR-432-5p) [44] that are closely correlated with tumor stage and poor survival [51]. Here, we found that E2F3 was a downstream target of hsa_Circ_0020256/miR-432-5p and involved in regulating CCA cell proliferation, migration, and invasion.

Due to their advantages of high stability and resistance to exonuclease degradation, CircRNAs are considered to be valuable biomarkers for use in diagnosing CCA and creating a prognosis for CCA patients [21]. Exosomal circRNAs serve as intercellular regulators of the tumorigenesis process [52]. Therefore, hsa_Circ_0020256 has potential future applications as a molecular target for use in diagnosing CCA and treating CCA patients. In conclusion, our data showed that TAM-secreted exosomes can significantly induce the proliferation, migration, and invasion of CCA cells both in vivo and in vitro. Circ_0020256 is delivered to CCA tumor cells by TAM-secreted exosomes, and then enhances the biological activity of the CCA cells. These increased cellular activities might be regulated by a Circ_0020256/miR-432-5p/E2F3 axis.

## Supplementary information

Supporting information

Supporting Table 5

Related manuscript file

## Data Availability

All data generated or analyzed during this study are included in this published article and its supplementary information files.
