# Supplementary material for: Tumor-associated macrophages promote cholangiocarcinoma progression via exosomal Circ_0020256
Source: Cell Death Dis. 2022 Jan 28;13(1):94. doi: 10.1038/s41419-022-04534-0 (PMC8799724; doi:10.1038/s41419-022-04534-0)
Supplement: Supplementary file 1 — Supporting information [file 41419_2022_4534_MOESM1_ESM.pdf]

Supporting Information

Supplementary Materials and Methods

Transfection

All siRNAs, miRNA mimic sequences (listed in **Supporting Table 2**), and plasmids used in the study were transfected into cells by using Lipofectemine 2000 according to the manufacturer's protocol. The cell treatments were as follows: RBE or HCCC-9810 cells were either co-cultured with exosomes (Exo) transfected with hsa_Circ_0020256 (Circ_X), or both treated with exosomes and transfected with hsa_Circ_0020256 siRNA (Exo+siCirc_X). RBE or HCCC-9810 cells were transfected with hsa_Circ_0020256 (Circ_X) or co-transfected with hsa_Circ_0020256 and the NC (Circ+NC) or miR-432-5p mimics (Circ_X+mimics). HEK293T cells were co-transfected with the wild type E2F3 (WT-E2F3) and NC or miR-432-5p mimics (mimics), or with the mutant form of E2F3 (MUT-E2F3) and NC or miR-432-5p mimics (mimics). RBE or HCCC-9810 cells were transfected with miR-432-5p mimics (mimics) or co-transfected with miR-432-5p mimics and the vector control (mimics+OE-NC) or E2F3 overexpression plasmid (mimics+OE-E2F3).

Flow cytometry

THP-1 cells were stimulated with PMA+IL-4 and collected by trypsinization. The cells were centrifuged at 300g for 5 min and then re-suspended in 200 μL of 1×PBS. Next, CD11b, CD163, CD80, and CD206 antibodies were added to the cells and incubated for 1 hour; after which, the cells were washed with 1×PBS and resuspended

in 500 μL of PBS. The fluorescence intensity was determined by flow cytometry.

CCK8 assay

RBE and HCCC-9810 cells were transfected with siRNA, miRNA mimics or the plasmid. After 24, 48, and 72 hours, 10 μL of CCK8 solution was added to each well and the cells were incubated for 2 hours. The absorbance increase was measured at 450 nm.

EdU staining

RBE and HCCC-9810 cells were transfected with siRNA, miRNA mimics or the plasmid. Next, 10 μM EdU was added to each well at 4 hours before fixation. After 15 mins of fixation, the cells were washed and permeabilized in 0.1% Triton X-100 for 20 mins. Edu solution buffer was then added to the cells, followed by incubation for 1 hour. The cell nucleus was stained with DAPI, and images were collected under a fluorescence microscope.

Wound healing assay

RBE and HCCC-9810 cells were seeded into 12 well plates and treated as indicated in the Figure legends. A scratch was made with a 200 μL pipette tip. Pictures were taken at the moment of scratching and 48 hours later. The cell migration rate was calculated as the distance between the scratches.

Transwell assay

RBE and HCCC-9810 cells were transfected with siRNA, miRNA mimics or plasmids. A total of 1 × 10^5 cells were placed in the upper chamber of a Transwell plate containing serum free medium. The bottom chamber contained 500 μL of cell culture medium. After 48 hours, cells which had not migrated through the membrane were removed, and cells which had migrated to the other side of the membrane were stained with crystal violet. Results were obtained from three independent experiments.

Western blotting

Tissues or cells were lysed with ice-cold RIPA lysis buffer and the total proteins were extracted. Next a 30 μg sample of total protein from each extract was separated by 10% SDS-PAGE, and the protein bands were transferred onto 0.2 μm PVDF membranes, which were subsequently blocked with 5% non-fat milk. The membranes were then incubated with anti-N-Cadherin (ab76011, Abcam, USA), E-Cadherin (ab40772, Abcam, USA), E2F3 (C03656B, SAB, USA), Histone H3 (ab1791, Abcam, USA), and GAPDH (ab8245, Abcam, USA) antibodies at 4°C overnight. On the next day, the membranes were washed with 1×TBST and incubated with an HRP-conjugated secondary antibody.

Transmission electron microscopy (TEM)

Exosomes were visualized and identified by using TEM as previously described. Briefly, exosomes were resuspended in PBS and fixed in 2% paraformaldehyde. The

fixed exosomes were dropped onto a carbon-coated copper grid, and subsequently stained with 2% uranyl acetate. Images were acquired with a JEM-1400 TEM operated at 80.0 kV.

Real-time PCR

Total RNA was extracted using Trizol reagent, and first stand cDNA was synthetized using a Bestar qPCR RT kit. The real time PCR system consisted of a 2×PCR mix and the appropriate primers (10 μM). The PCR conditions were as follows: 94°C for 20 s, 58°C for 20 s, and 72°C for 20 s, for a total of 40 cycles. The data were analyzed using the $2^{-\Delta\Delta CT}$ method. The primer sequences used are listed in **Supporting Table 3**.

Target gene prediction

miRanda (http://www.microrna.org/) was used to predict potential targets of Circ_0020256. TargetScan (http://www.targetscan.org/) and Starbase (https://starbase.sysu.edu.cn/index.php) were used to predict the potential target gene of hsa-miR-432-5p. As predicted, the 3' UTR of E2F3 contained the binding sites for hsa-miR-432-5p.

Luciferase assay

The 3' UTR of E2F3 containing the putative binding sites for miR-432-5p or the full length of hsa_Circ_0020256 containing putative binding sites for miR-432-5p was

amplified using the primers listed in **Supporting Table 3.** It was then subcloned into a psiCHECK-2 vector and confirmed by sequencing. To perform the dual-luciferase assay, cells were seeded onto a 96-well plate and co-transfected with miR-432-5p or miR-NC and the wild-type or mutant form of Circ_0020256 or E2F3. After 48 hours, luciferase activity was examined using a Dual-Luciferase® Reporter Assay System (Promega, Madison WI, USA).

MiRNA Target IP

HCCC-9810 or RBE cells (5×10^6 cells in a 15 cm dish) were transfected with the NC or miR-432-5p mimics. After 24 hours of transfection, the cells were collected and lysed in IP lysis buffer. The miRNA Target IP assay was performed using a commercial kit (Active motif, Cat. No. 25500). Briefly, 50 μL of Protein G beads plus 200 μL of BSA solution were added to cells and then incubated for 10 min at room temperature. Next, the incubation tubes were placed on a magnet to pellet the beads, and the beads were washed with 1×wash buffer. Next, 5 μg of antibody (Ago2) was added and incubated for 30 min at room temperature. IgG was used as a negative control. The RNA complex immunoprecipitate was centrifuged, washed, and digested with proteinase K. The RNA was purified and hsa_Circ_002056 expression was detected by real-time PCR.

H&E staining

The xenograft tumors or CCA tissues were fixed with 4% paraformaldehyde and

embedded in paraffin. Next, the slide-mounted tissues were dewaxed with xylene, rehydrated with a gradient ethanol series, gently washed three times with PBS, and then stained with hematoxylin and eosin. The stained sections were examined under a light microscope (Olympus, Tokyo, Japan) at magnifications of x20 and x40, respectively.

Immunohistochemistry

Tumors or CCA tissues were fixed with 4% paraformaldehyde and embedded in paraffin blocks. Next, the slide-mounted tissues were dewaxed with xylene, rehydrated with a gradient ethanol series, gently washed three times with PBS, and then stained with Anti-E2F3 (C03656B, SAB, USA) or Anti-Ki67 (ab16667, Abcam, USA) antibody for 1 h at 37°C. After being washing three times with PBS, the tissues were incubated with HRP-conjugated secondary antibodies for 1 h at room temperature. The cell nuclei were counterstained with hematoxylin for 5 min.

Fluorescence in situ hybridization (FISH)

Control or CCA tissues were fixed and embedded with OTC. After prehybridization and hybridization, the slide-mounted tissues were incubated overnight with a Cy3-labelled hsa_circ_0020256 probe at 37°C. Images of the tissues were acquired with a ZEISS LSM800 Confocal Microscope (Carl Zeiss AG, Germany).

Supporting Figure legends

Supporting Fig. 1. TAMs promoted the migration of RBE and HCCC-9810 cells.

RBE and HCCC-9810 cells were co-cultured with M2 macrophages (M2), untreated THP-1 cells (Un-mac), or treated with TGF-β. The wound healing results are shown.

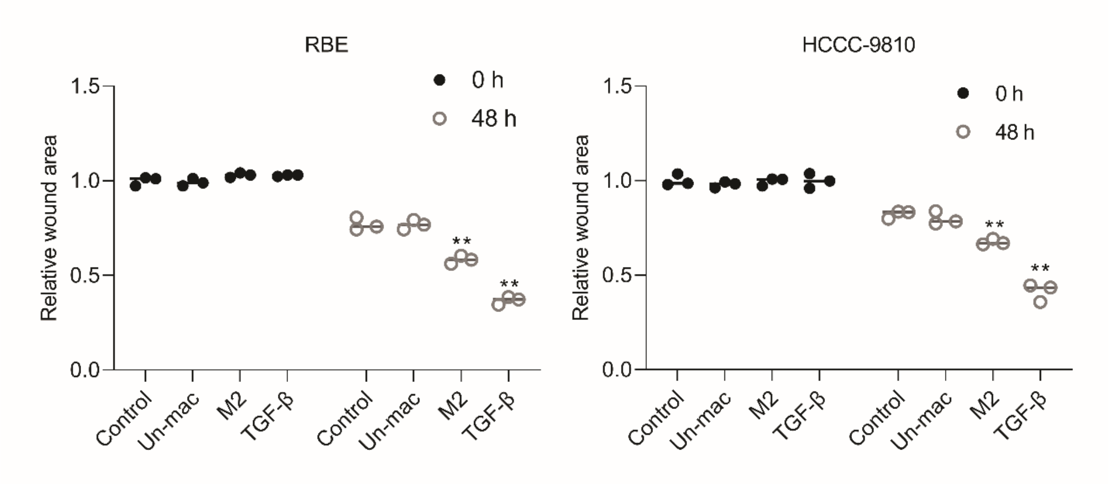

Supporting Fig. 2. Identification of exosomes in the CM of TAMs. (A) A typical transmission electron microscopy picture is shown. **(B)** The identification of exosomes was confirmed by western blotting. **(C)** RBE and HCCC-9810 cells were co-cultured with exosomes (Exo) or treated with TGF-β. The wound healing results are shown.

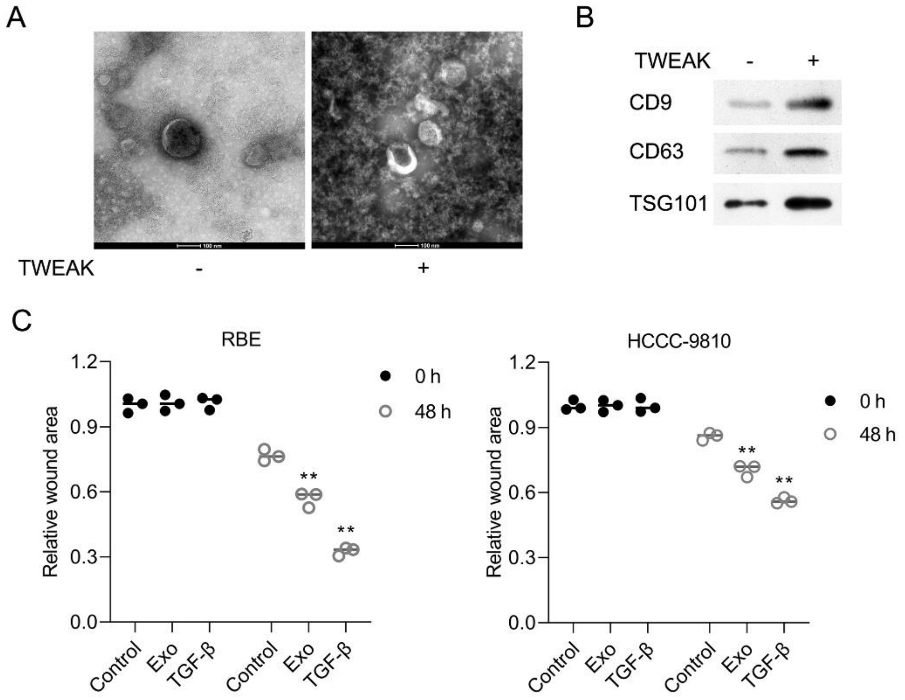

Supporting Fig. 3. Hsa_Circ_0020256 promoted the proliferation, migration, and invasion of CCA cells by sponging miR-432-5p. (A) HCCC-9810 cells were transfected with hsa_Circ_0020256 (Circ_X) or co-transfected with hsa_Circ_0020256 and the NC (Circ+NC) or miR-432-5p mimics (Circ_X+mimics). Hsa_Circ_0020256 and miR-432-5p expression were detected by real-time PCR. **(B)** CCK8 assay results for HCCC-9810 cells are shown. **(C)** The respective images of Edu staining results and a summary of results from three independent experiments are shown. **(D)** Cells monolayers were wounded and images were acquired at 0, and 48 hours. The respective images of Transwell assays are shown. **(E)** Images of Transwell assays and summary results of Transwell assays are shown. **, $p < 0.01$ compared with the Circ-NC group. #, $p < 0.05$ and ##, $p < 0.01$ compared with the Circ_X+NC group.

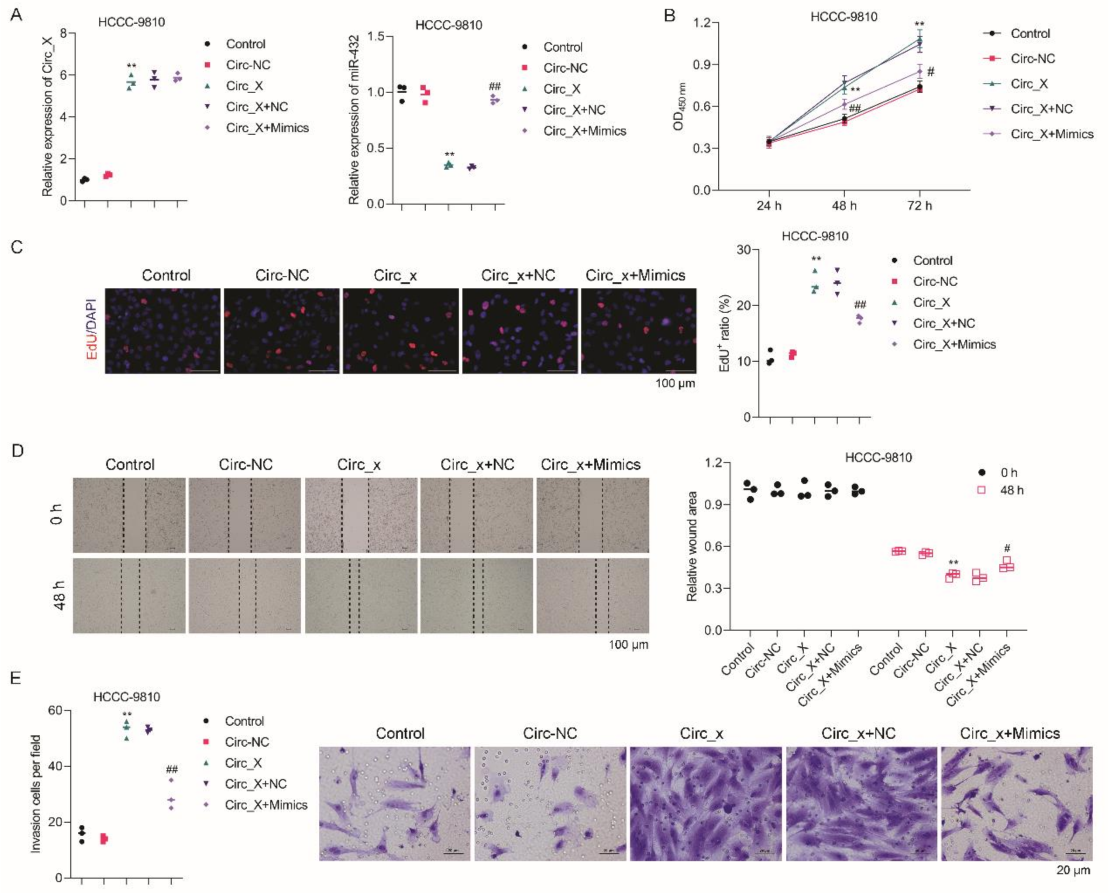

Supporting Fig. 4. MiR-432-5p and E2F3 expression. HCCC-9810 cells were transfected with hsa_Circ_0020256 (Circ_X) or co-transfected with hsa_Circ_0020256 and the NC (Circ+NC) or miR-432-5p mimics (Circ_X+mimics). **(A)** E2F3 expression was detected by real-time PCR. **(B-C)** The levels of E2F3, N-Cadherin, and E-Cadherin protein expression were determined by IF or western blotting. **, $p < 0.01$ compared with the Circ-NC group. ##, $p < 0.01$ compared with the Circ_X+NC group.

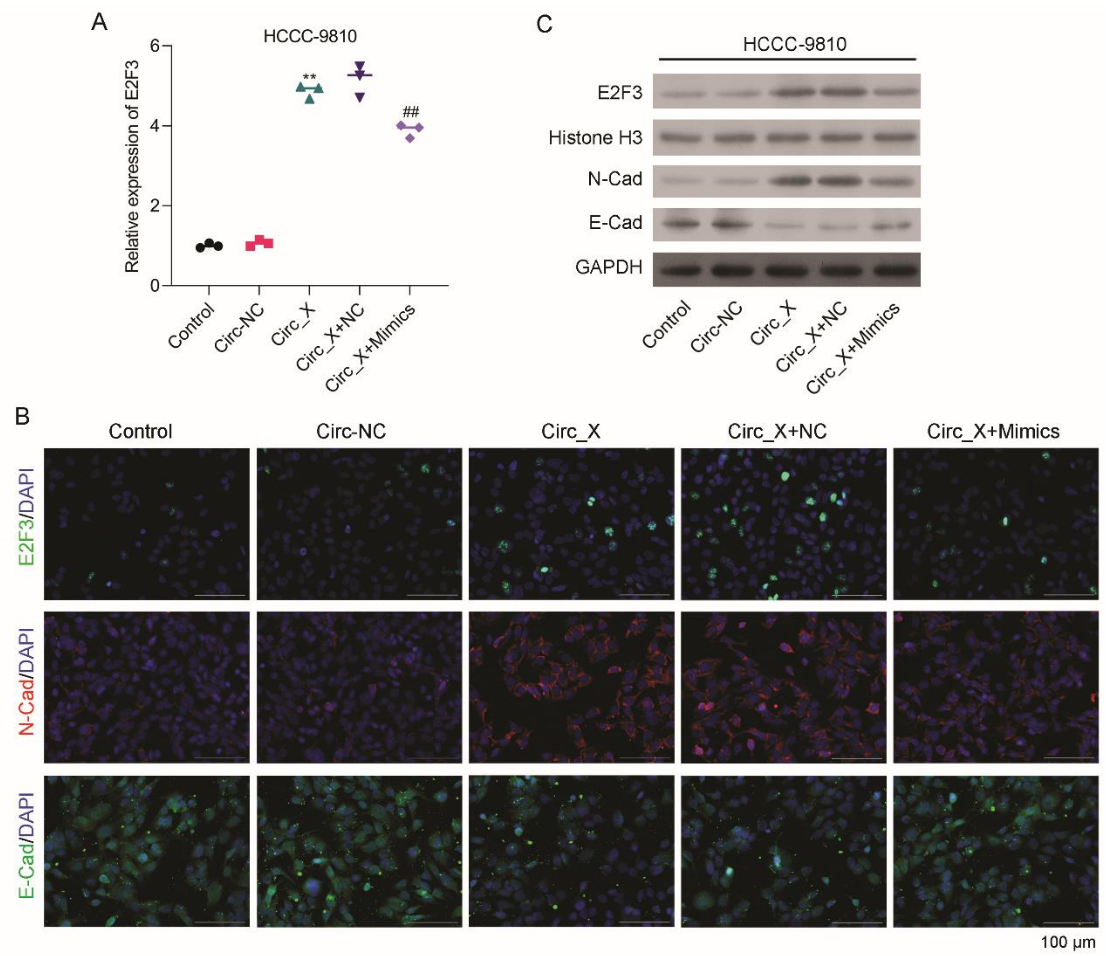

Supporting Fig. 5. Exosomal Hsa_Circ_0020256 mediated TAM-induced tumor progression *in vivo* via the miR-432-5p/E2F3 axis. HCCC-9810 cells (1 x 10^7) were subcutaneously injected into nude mice. **(A)** The nude mice were sacrificed after 4 weeks. Images of the xenografts are shown. **(B)** Tumor volumes were calculated 3, 7, 14, 21 and 28 days after subcutaneous injection of cell. **(C)** Xenograft tumor tissues were collected and the RNA was extracted. Hsa_Circ_0020256, miR-432-5p, and E2F3 expression were detected by real-time PCR. **(D)** E2F3, Histone H3, N-Cadherin, and E-cadherin protein expression in xenograft tumor tissues was detected by western blotting. **(E)** Tumor tissues were collected and fixed for H&E staining and IHC performed using Ki67 and E2F3 antibodies.

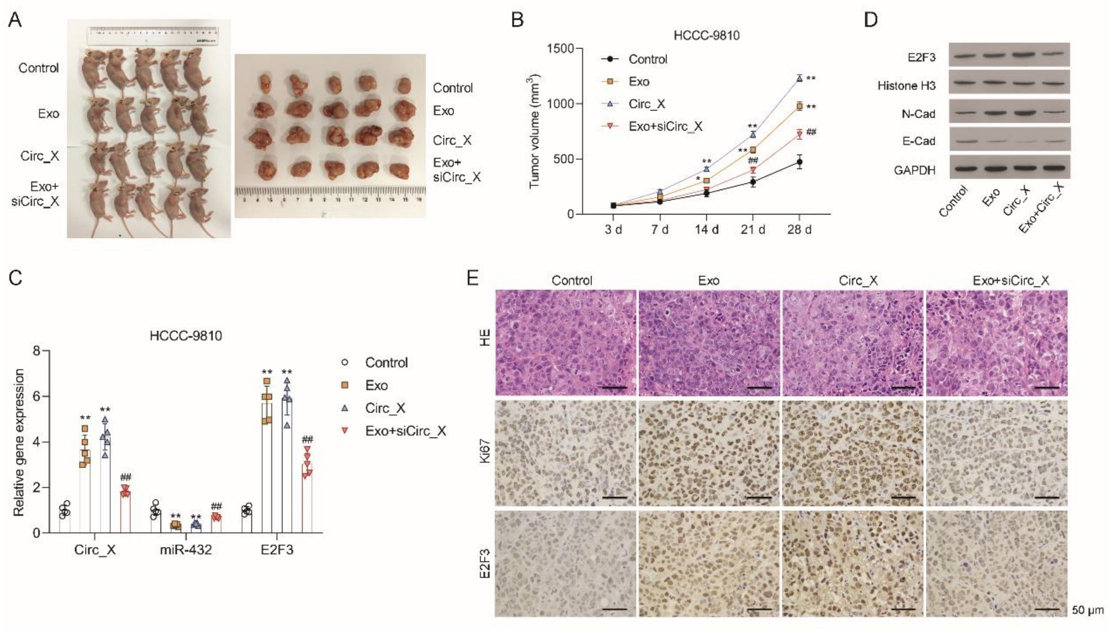

Supporting Fig. 6. Circ_0020256 expression in CCA patients and the relation between Circ_0020256 and overall survivial. (A) Thirty pairs of CCA tissue and adjacent tissue were collected and analyzed by real-time PCR to detect Circ_0020256 expression. ***, $p < 0.001$. (**B**) The relationship between Circ_0020256 expression and the overall survival of CCA patients was summarized by a correlation analysis. (**C**) The relationship between Circ_0020256 expression and the recurrence time of CCA patients was summarized by a correlation analysis.

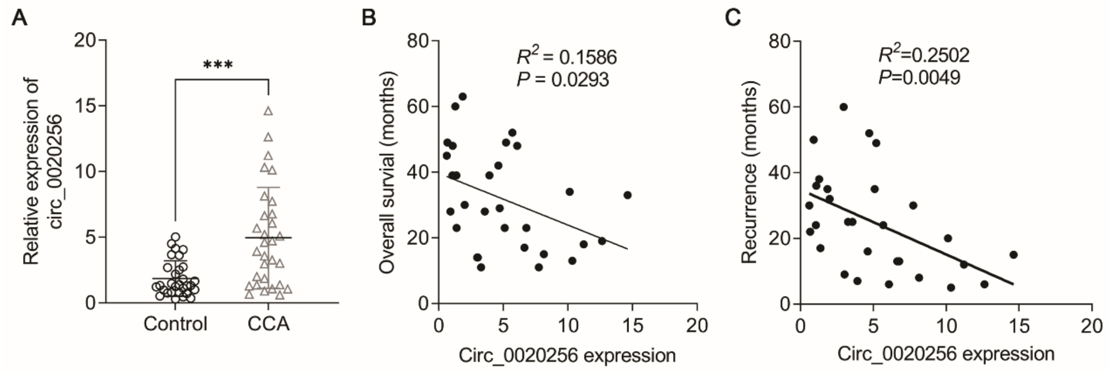

Supporting Tables

Supporting Table 1. Clinical information for the human cholangiocarcinoma specimens.

Parameter	All patients (n = 30)
Gender	
Male	19 (63.33)
Female	11 (36.67)
Age	
< = 60	16 (53.33)
> 60	14 (46.67)
Type	
hCCA	19 (63.33)
dCCA	11 (36.67)
Vascular invasion	
Yes	20 (66.67)
No	10 (33.33)
Lymph node metastasis	
Yes	30 (100)
No	0 (0)
Differentiation	
Low	11 (36.67)
Medium	8 (26.67)
High	11 (36.66)
T stage	
T1	0 (0)
T2	0 (0)
T3	18 (60)
T4	12 (40)

hCCA, hilar cholangiocarcinoma; dCCA, distal cholangiocarcinoma.

Supporting Table 2. SiRNA and miRNA mimic sequences used in the study.

ID	Sense (5’-3’)	Antisense (5’-3’)
Negative control	UUCUCCGAACGUGUCACGUTT	ACGUGCCACGUUCGGAGAATT
siCirc_0020256	GGCAGACAAUAAUGAACAUUU	UUCCGUCUGUUAUUACUUGUA
miR-432-5p mimics	GGUGGGUUACUGGAUGAGGUUCU	AACCUCAUCCAGUAACCCACCUU

Supporting Table 3. Primers used in the study.

ID	Sequence (5’- 3’)
GAPDH F	TGTTCGTCATGGGTGTGAAC
GAPDH R	ATGGCATGGACTGTGGTCAT
E2F3 F	GCACTACGAAGTCCAGATAG
E2F3 R	TTAATGAGGTGGATGCCTTC
U6 F	CTCGCTTCGGCAGCACA
U6 R	AACGCTTCACGAATTTGCGT
All R	CTCAACTGGTGTCGTGGA
hsa-miR-432	TCTTGGAGTAGGTCATTGGGTGG
hsa-miR-432 RT	CTCAACTGGTGTCGTGGAGTCGGCAATTCAGTTGAGCCACCCA
hsa-miR-432 F	ACACTCCAGCTGGGTCTTGGAGTAGGTCATTGG
hsa_circ_0000772-F	GAGGAAATGGTGGCTCAGAGTGTGT
hsa_circ_0000772-R	GTTTTGAGTTTTCAACATGCCATCC
hsa_circ_0007357-F	CGCACCCCTGCACTACTATGAGACC
hsa_circ_0007357-R	CTGCCCTGCCTCCCTATCTGCA
hsa_circ_0012782-F	AACAAGTTTTAGAAGACAGTCCAGC
hsa_circ_0012782-R	CAATACTGATCCCAAGAGACACATT
hsa_circ_0017992-F	TGAAATGAAATGATGGCCACG
hsa_circ_0017992-R	AAATCCGAATGGGTCGTGAA
hsa_circ_0020256 F	CCGTGACTGGGAGGAGATT
hsa_circ_0020256 R	ACTGTACGGGGGAAAGAATG

hsa_circ_0031017-F	GTCATGCCGACTCTCATACA
hsa_circ_0031017-R	TCCCAACACCTTCATGATCC
hsa_circ_0042937-F	GTCATGCCGACTCTCATACA
hsa_circ_0042937-R	TCCCAACACCTTCATGATCC
hsa_circ_0052132-F	TGCCTTCGCCGCTTCCTCC
hsa_circ_0052132-R	CGGATGTGGCCGATGTTGCTG
hsa_circ_0057974-F	CGAACACCTGTTGGGAGTAG
hsa_circ_0057974-R	GTGTGGGTGACCTAACAGAC
hsa_circ_0062300-F	GGAAAGGCCGCCCACTCAGTG
hsa_circ_0062300-R	GCGTCTCTATGATCCTGGCTTCTGG
hsa_circ_0062531-F	CCGAAAACAGACAGGGGTC
hsa_circ_0062531-R	GAGTGTGCTTCAGCAAGTCAT
hsa_circ_0065492-F	TACCTAGAGGAGCTGCTGCATATTC
hsa_circ_0065492-R	GGAGGGCATGGTGTTGTGG
hsa_circ_0074800-F	GCTGACGACACCTGCCTGG
hsa_circ_0074800-R	CTCTAGCCGTAGGGCAGTCATAGTC
hsa_circ_007975-F	ACAGCAAGCACAAAAATCCTTACC
hsa_circ_007975-R	CACTGTGGCTGCATATTCCAAATTC
hsa_circ_0079753-F	ATGAACTACTCACACGTTATGATCTG
hsa_circ_0079753-R	GGCTGCATATTCCAAATTCTTC
hsa_circ_0082491-F	GTTCTTACGCTGGTGTCACAGATTG
hsa_circ_0082491-R	ATAGACTAGCAGCCGAATTTACCGC
hsa_circ_0083135-F	CAAGAGAAAACTTACAATCCCTTCTATG
hsa_circ_0083135-R	AAGGCAGCAATCCATGAGAAC
hsa_circ_0084335 -F	GGAGGTGGTACTTTGTCGTG
hsa_circ_0084335 -R	TGTTTCGCAACCAGTTCACA
hsa_circ_0091577 -F	TTCCTGGAAACCCACCTCTTG
hsa_circ_0091577 -R	TAAGGATTGAAAGCCATGGGAG
hsa_circ_0092351-F	ACCATGAGTGCCGGCCTTGT

hsa_circ_0092351-R	CCAGACAGCCCCACCCAC
E2F3-MUT-F	CTTCCTACCTTCTTCGATGCTACGAGTATCATGAAGTAAAC
E2F3-MUT-R	GTTTACTTCATGATACTCGTAGCATCGAAGAAGGTAGGAAG
circ_0020256-MUT-F	GATTTCAGAGCCTGTGATCTGCTAGCCTCAGAGGCAGCAG
circ_0020256-MUT-R	CTGCTGCCTCTGAGGCTAGCAGATCACAGGCTCTGAAATC

Supporting Table 4. CircRNAs that were differentially expressed between the control and test groups.

File name	Threshold		Number of Differentially Expressed Genes		
	P-value threshold	Fold-change threshold	Number of differentially expressed CircRNAs	Number of up-regulated CircRNAs	Number of down-regulated CircRNAs
Test_vs._Control_p001fc2	0.01	2	524	169	355
Test_vs._Control_p001	0.01		1986	965	1021
Test_vs._Control_p005fc2	0.05	2	1284	495	789
Test_vs._Control_p005	0.05		5927	3213	2714

A total of 5927 differentially expressed circRNAs were found in test group when compared with the control group ($p < 0.05$, abbreviated as p005). Among those differentially expressed circRNAs, 3212 circRNAs were up-regulated and 2714 circRNAs were down-regulated (p005). A total of 1284 differentially expressed circRNAs had changes > 2-fold (abbreviated as fc2), Among those circRNAs, 495 circRNAs were up-regulated and 789 circRNAs were down-regulated (p005). A total of 1986 differentially expressed circRNAs were found in the test group when compared with the control group ($p < 0.01$, abbreviated as p001). Among those circRNAs, 965 circRNAs were up-regulated and 1201 circRNAs were down-regulated (p001). A total of 524 of the differentially expressed circRNAs had changes > 2-fold (fc2), Among those circRNAs, 169 circRNAs were up-regulated and 355 circRNAs were down-regulated (p001).

Supporting Table 5. List of differentially expressed CircRNAs in control and test

groups ($p<0.05$, fold change>2).
